# Differential effects of antibiotics on neutrophils exposed to lipoteichoic acid derived from *Staphylococcus aureus*

**DOI:** 10.1186/s12941-020-00392-w

**Published:** 2020-11-03

**Authors:** Marquerita Algorri, Annie Wong-Beringer

**Affiliations:** University of Southern California, School of Pharmacy, 1985 Zonal Avenue, Los Angeles, CA 90089 USA

**Keywords:** Antibiotics, Immunomodulation, *Staphylococcus aureus* bacteremia, Lipoteichoic acid, Ceftaroline

## Abstract

**Background:**

Persistent bacteremia occurs in at least 30% of patients with *Staphylococcus aureus* bloodstream infection (SAB) and may be attributable to a dysregulated host immune response. Neutrophils interact with a variety of *S. aureus* microbial factors, including lipoteichoic acid (LTA), to activate phagocytic function in a concentration-dependent manner. Antibiotics have been shown to exert both direct antimicrobial action as well as immunomodulatory effects. In this study, we compared the effects of different anti-staphylococcal antibiotics on LTA-mediated immune activation of neutrophils.

**Methods:**

Neutrophils obtained from healthy volunteers were exposed to two levels of LTA (1 and 10 μg/ml) with or without addition of antibiotics from different pharmacologic classes (vancomycin, daptomycin, ceftaroline). Neutrophil function was assessed by examining phagocytic response, activation (CD11b, CD62L expression), Toll-like receptor-2 expression, cell survival and apoptosis, and CXCL8 release.

**Results:**

Differential LTA-mediated antibiotic effects on neutrophil function were observed primarily at the high LTA exposure level. Ceftaroline in the presence of 10 μg/ml LTA had the most prominent effects on phagocytosis and CD11b and CD62L expression, with trends towards increased neutrophil survival and preservation of CXCL8 release when compared to daptomycin and vancomycin with the latter significantly dampening PMN CXCL8 release.

**Conclusions:**

Select antimicrobial agents, such as ceftaroline, exert immunostimulatory effects on neutrophils exposed to *S. aureus* LTA, which when confirmed in vivo, could be leveraged for its dual immunomodulatory and antibacterial actions for the treatment of persistent SAB mediated by a dysregulated host response.

## Introduction

*Staphylococcus aureus* is a leading cause of bacteremia and gram-positive sepsis in the United States, affecting 80 per 100,000 individuals annually [1, 2]. We and others have shown that persistent bacteremia (SAB) despite receipt of antibiotics with in vitro activity, affects approximately 1 in 3 patients and is a strong risk factor for mortality [3–7]. Specifically, each day of persistence causes a 16% increase in the risk of 30-day mortality [7]. Recent evidence points to a dysregulated host immune response contributing to the development of persistence [3, 8–10]. *S. aureus* possesses a variety of virulence factors that can contribute to immune dysfunction by modulating targeted pro-inflammatory responses. Importantly, *S. aureus* can subvert the phagocytic actions of neutrophils (PMNs), which has been demonstrated in animal models to drive persistent infection [11, 12].

Of interest, during *S. aureus* infection, neutrophils respond to a variety of bacterial pathogen associated molecular patterns (PAMPs), including peptidoglycan and lipoteichoic acid (LTA), which are recognized principally by the pathogen recognition receptor (PRR), Toll-like Receptor 2 (TLR2) [13]. LTA is a lipoprotein cell wall component of all gram-positive bacteria that is released from the bacterial cell wall spontaneously or following exposure to antibiotics [14, 15]. Previous studies have reported that LTA has significant pro-inflammatory effects on innate immune cells and is able to initiate neutrophil activation, TLR2 signaling, and pro-inflammatory cytokine response [15–17]. Upon activation, PMNs undergo their effector functions that aim to kill invading pathogens, including phagocytosis of bacteria; chemotaxis and migration to the site of infection; interleukin-8 (CXCL8) secretion, which recruits additional PMNs and immune cells; and prolonged lifespan, as evidenced by delayed apoptosis [18, 19]. Activation can be detected by shedding of L-selectin (CD62L) from the cell surface and upregulation of integrin alpha M (CD11b) expression [16, 20]. Ectodomain shedding of CD62L is necessary for PMN “rolling” along epithelial tissues during chemotaxis, whereas CD11b is needed for PMN adhesion to endothelial cells at the site of infection [21].

Baseline release of LTA from clinical strains that caused bloodstream infections appears to vary by 14-fold (unpublished data). In addition, exposure to antibacterial agents appears to differentially affects the release of LTA release from *S. aureus.* Specifically, exposure to cell-wall active antimicrobial agents, such as imipenem, flucloxacillin, and cefamandole has been shown to significantly enhance the release of LTA whereas exposure to protein synthesis inhibiting drugs, including clindamycin, gentamicin, and erythromycin, inhibits LTA release [14, 15]. Therefore, we hypothesized that unique classes of anti-staphylococcal antibiotics may have differential capacities for stimulating LTA-mediated PMN response. In this study, we sought to investigate the stimulatory effects of ceftaroline, which has been used successfully as salvage therapy in persistent MRSA bacteremia [22]. We examined the immunomodulatory potential of ceftaroline compared to the standard treatment vancomycin and daptomycin in the presence of LTA. We tested antibiotics under the conditions of a high and a low exposure level of LTA in human PMNs, to model the scenario in which different amounts of LTA are released during infection due to variations in clinical strains and to examine the differential immunomodulatory potential of antibiotics. Our results indicate that the pro-inflammatory effect of LTA is concentration-dependent and that ceftaroline strongly promotes PMN activation and phagocytosis while preserving cell survival and CXCL8 release, relative to other commonly used drugs, such as vancomycin, which impairs CXCL8 secretion. In the context of persistent bacteremia, our findings suggest that ceftaroline may be a preferred agent for stimulating PMN function to facilitate prompt bacterial clearance and deserves confirmation in vivo.

## Materials & methods

### Isolation of human polymorphonuclear leukocytes (PMNs) from healthy donors

PMNs were obtained from whole blood of healthy volunteer donors (n = 5). All donors gave informed consent to participate in the study, as approved by the Institutional Review Board (IRB) for the University of Southern California Health Sciences Campus. All research in this study was conducted in compliance with IRB-approved methods and international human-subjects research regulations.

PMNs were isolated from whole blood using EasySep™ Direct Human Neutrophil Isolation Kit (STEMCELL Technologies, Vancouver, Canada). Briefly, whole blood was diluted and incubated with immunomagnetic antibodies that bind to unwanted cells, leaving PMNs undisturbed and in solution upon application of the tube to the EasySep™ magnet (STEMCELL Technologies, Vancouver, Canada). Isolated cells were washed in PBS thrice and seeded at a concentration of 1 × 10^6^ cells/ml in RPMI 1640 medium and 10% FBS at 37 °C in 5% CO_2_. PMN purity following isolation ranged from 97–99%, as determined via FSC and SSC flow cytometric analysis as well as microscopic analysis using Kwik-Diff™ staining (Fisher Scientific, Waltham, MA). Following isolation, cells were stimulated as indicated below.

## Cell culture and stimulation

### Stimulation conditions

Cells were either unstimulated (cell culture media alone), stimulated with antibiotics alone, stimulated with 1 μg/ml or 10 μg/ml of commercially obtained purified LTA from *S. aureus* (Invivogen, San Diego, CA, USA), or stimulated with 1 or 10 μg/ml of LTA plus antibiotics. Antibiotics and LTA were added to the cells simultaneously. The following anti-staphylococcal agents were selected for testing to represent different pharmacologic classes: vancomycin (VAN), daptomycin (DAP), and ceftaroline (CFT). Clinical formulations of Cubicin (daptomycin) (Merck & Co., Inc, Kennilworth, NJ) and Vancomycin (Hospira Inc, Lake Forest, IL) were obtained from a hospital clinical pharmacy unit. As ceftaroline fosamil used in the clinical setting is a pro-drug formulation, the active form of CFT was provided by the manufacturer (Allergan, Dublin, Ireland) in this study. Antibiotics were tested at concentrations needed to achieve the target AUC/MIC24 (VAN, DAP) or 5 × MIC (CFT) for efficacy using the respective MIC for each drug against the common and well-characterized LAC USA300 community-acquired MRSA clinical strain [23–25]. MICs were determined using broth microdilution testing and E-Test (bioMérieux, Marcy-l’Étoile, France). Concentrations of antibiotics used were 12.5 μg/ml, 1.25 μg/ml, and 3.29 μg/ml, for VAN, CFT, and DAP, respectively.

### Timepoint measurement

The timepoints at which phagocytosis, flow cytometry, cell lifespan, and cytokine measurements were taken are described below. Phagocytosis studies were completed following 4 h of stimulation with LTA, as preliminary optimization studies indicated that PMN stimulation beyond 4 h exhibited a diminished phagocytic response, likely due to the rapid effector action of activated PMNs. Examination of CD11b and CD62L was conducted at 4 h which is consistent with previously conducted studies showing sufficient and differential expression of CD11b and CD62L with LPS at 3 h while others have assayed PMN CD11b expression at 6 h [16, 26]. Therefore, we have selected the 4 h timepoint for measurement of phagocytosis and cell surface expression of CD11b and CD62L as well as TLR-2 to allow for comparison between phagocytosis and activation data. CXCL8 cytokine measurements were collected at 16 h, to compensate for both peak release and degradation. Previous studies have also shown optimal CXCL8 release in PMNs with LTA stimulation at 16 h [15, 16]. Apoptosis and cell survival was measured at 16 h via flow cytometry since previous studies have shown that 40–60% of unstimulated PMNs are apoptotic between 12–20 h [27, 28]; thus, we chose the later timepoint within the range to allow for sufficient capturing of the effect of LTA on PMN survival.

### PMN functional assessment by flow cytometry analysis

Following stimulation with LTA and/or antibiotics, PMNs were harvested for flow cytometry analysis. At 4 h, CD11b, CD62L, and TLR2 expression was analyzed by staining cells in separate tubes with Rat PE Anti-Human CD11b Clone M1/70 (BD Biosciences, San Jose, CA, USA), Mouse FITC Anti-Human CD62L clone DREG-56 (ThermoFisher Scientific, Waltham, MA, USA), and PE Mouse Anti-Human CD282 (TLR2) Clone: TL2.1 (ThermoFisher Scientific, Waltham, MA, USA). At 16 h, PMN apoptosis was assessed by labeling PMNs with the Invitrogen Alexa Fluor® 488 Annexin V/Dead Cell Apoptosis Kit, per manufacturer’s instructions (ThermoFisher Scientific, Waltham, MA, USA). Data was collected using the BD LSR Fortessa X-20 and analyzed using FlowJo software (FlowJo, LLC, Ashland, OR, USA). Cells were gated initially using FSC and SSC parameters to remove debris and dead cells; however, in the analysis of cell lifespan and apoptosis, dead cells, as marked by PI, were not gated out. Unstained controls, as well as single-stained cells, were used to compensate for background and spillover fluorescence for all experiments in which more than 1 stain was used (CD11b/CD62L; FITC annexin V and PI). Corresponding isotype controls were also utilized to optimize antibody concentrations used. A minimum of 10,000 cells per condition were analyzed for all flow cytometry experiments.

### Analysis of PMN phagocytosis

Phagocytic activity was assessed after incubating PMNs at 37 °C for 3 h in a 96-well flat-bottom plate with or without LTA and/or antibiotics, added simultaneously. Following 3 h of incubation in the presence of LTA and/or antibiotics, the pHrodo green *S. aureus* Bioparticles Conjugate for Phagocytosis (ThermoFisher Scientific, Waltham, MA, USA) was added to assess for changes in pH associated with phagocytosis per manufacturer’s instructions. PMNs were incubated with pHrodo for 1 h at 37 °C without CO_2_ before reading on a spectrofluorometer, for a total incubation time of 4 h (3 h LTA/antibiotics followed by 1 h LTA/antibiotics with added pHrodo).

### Cytokine quantification by ELISA

Cell culture supernatants were collected in triplicate from PMNs following 16 h of stimulation with antibiotics and LTA. Supernatants were stored at − 80 °C until use. Release of CXCL8 was determined using MesoScale Discovery multiplex ELISA and analyzed in duplicate (MesoScale Discovery, Gaithersburg, MD, USA). The accompanying SECTOR Imager SI2400 and MSD Workbench software were used for analysis. The lower limit of detection for CXCL8 was 0.16 pg/mL, as per manufacturer’s instructions. Samples with values below the limit of detection were assigned a value of 0 pg/ml.

### Statistical analysis

Statistical analysis was performed using Graphpad Prism version 8.0 (Graphpad Software, San Diego, CA, USA) Data are represented through mean and standard error. Two-way ANOVA with Tukey’s multiple comparisons test was utilized to assess statistical differences between treatment groups. Correlation analysis was performed by calculating Pearson’s r correlation coefficients. P values ≤ 0.05 were considered significant.

## Results

### Neutrophil activation, phagocytosis, and cell survival is differentially affected by LTA exposure level and antibiotics

We used two different exposure levels of LTA (high: 10 μg/ml; low: 1 μg/ml) in combination with anti-staphylococcal antibiotics (vancomycin, VAN; daptomycin, DAP; and ceftaroline, CFT) to stimulate PMNs isolated from healthy human donors. To measure whether antibiotics promote neutrophil activation, we assessed cell surface expression of CD11b and CD62L after 4 h of stimulation with LTA and antibiotics. During PMN activation, CD11b has an inverse relationship with CD62L; while CD11b is upregulated, CD62L is shed from the cell surface [16, 20, 21].

**Fig. 1:**
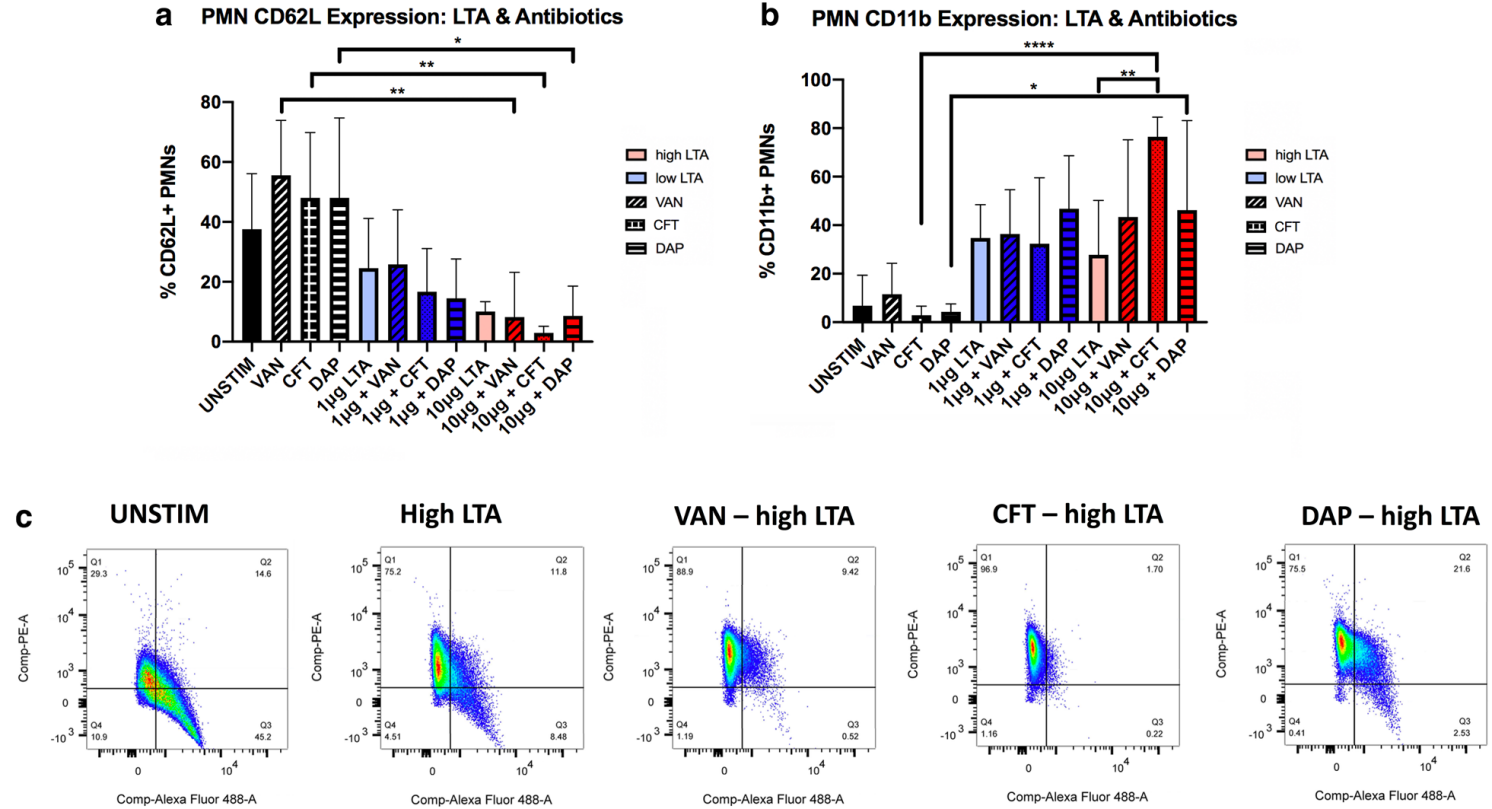
Effects of high (10 μg) and low (1 μg) level of LTA and antibiotics on CD11b (**a**) and CD62L expression (**b**) in human neutrophils isolated from healthy volunteers (n = 5). CD11b and CD62L expression were determined at 4 h following stimulation using flow cytometry. CD62L and CD11b are inversely correlated markers of PMN activation, with CD62L shedding and CD11b upregulation indicative of activation. Representative scatter plots of unstimulated cells and cells exposed to high LTA with and without antibiotics are shown in (**c**). Statistical significance was determined using Two-way ANOVA * p < 0.05; **p < 0.01. VAN, vancomycin; CFT, ceftaroline; DAP, daptomycin

PMNs exposed to high level of LTA appeared to show increased activation in the presence of all antibiotics (Fig. 1a–c). Compared to high LTA alone, addition of CFT robustly induced CD11b expression and decreased CD62L expression in PMNs, indicating a greater proportion of activated PMNs in the presence of CFT (CD11b, p = 0.01; CD62L, p = 0.03). There was no significant difference between high and low LTA effects on CD11b and CD62L expression in the absence of antibiotics (CD11b, p = 0.99; CD62L, p = 0.95). At the lower level of LTA, none of the antibiotics tested had a significant effect on CD11b or CD62L expression. DAP and VAN modestly increased CD11b and decreased CD62L, but the effect was not significant. Antibiotics alone did not appear to have significant effects on CD11b or CD62L, though a small increase in CD62L can be observed with the addition of antibiotics without LTA, indicating a tendency for PMNs to remain in the resting state with antibiotics alone. In addition to its role in inflammation, CD62L is also a marker of neutrophil age [29]. As antibiotics also had a small effect on increasing PMN longevity, as discussed in further detail below, it is possible that this antibiotic-mediated change in longevity is associated with a decreased tendency for PMNs to progress towards the “aged” phenotype, which may be associated with antibiotic anti-inflammatory action.

### Neutrophil phagocytosis

**Fig. 2:**
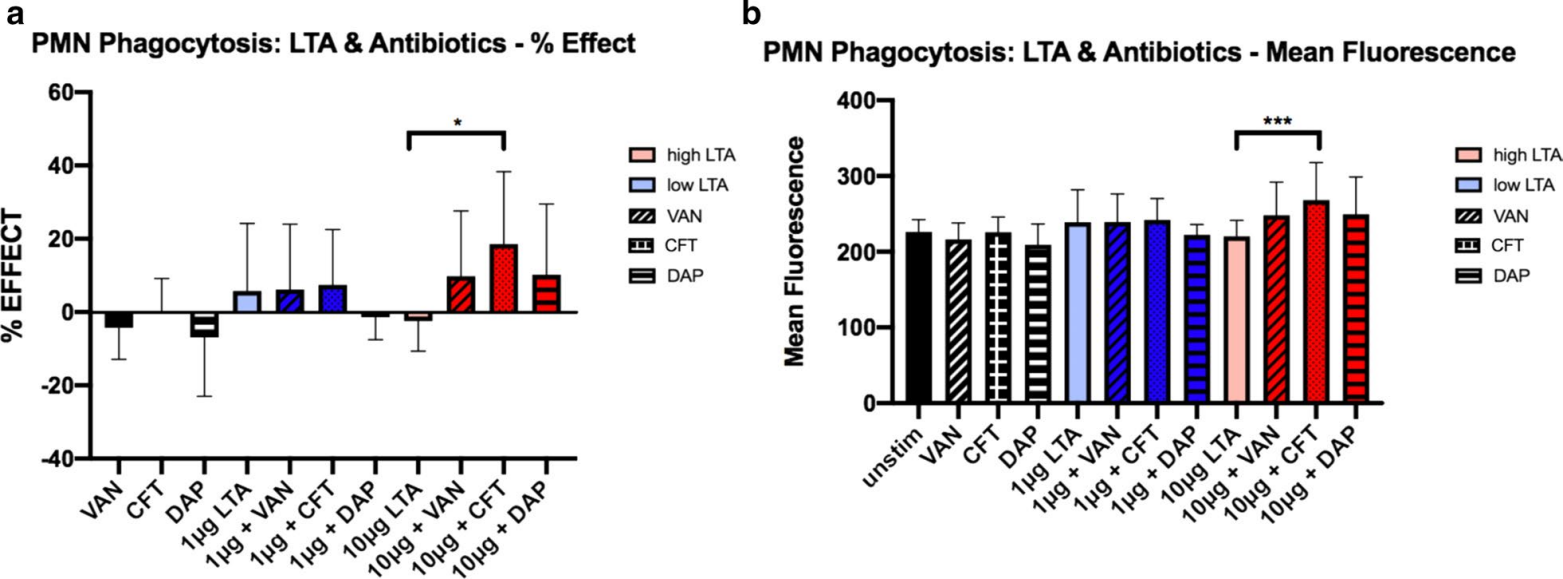
Effects of high (10 μg) and low (1 μg) levels of LTA and antibiotics on phagocytosis in human neutrophils isolated from healthy volunteers (n = 5). Phagocytosis was assessed at 4 h after stimulation using pHrodo *S. aureus* bioparticles and a fluorescent plate reader. Percent effect, based on the unstimulated control is shown in (**a**). Mean fluorescence values, as determined via fluorescence spectrophotometry, are depicted in (**b**). Statistical significance was determined using Two-way ANOVA * p < 0.05. VAN, vancomycin; CFT, ceftaroline; DAP, daptomycin

Human PMNs exposed to high and low levels of LTA in the presence of antibiotics were analyzed for phagocytic function following 4 h of stimulation. Phagocytosis was assessed using pHrodo *S. aureus* bioparticles, a commercially available fluorescent pH-dependent assay which produces more fluorescence when particles are phagocytosed due to an alteration in acidity. CFT alone had no effect on phagocytosis while VAN and DAP had non-significant inhibitory effects (Fig. 2a, b). LTA at the high exposure level alone caused a slight decrease in phagocytosis, but this observed effect was reversed by all tested antibiotics. In particular, addition of CFT to PMNs exposed to high LTA level significantly increased PMN phagocytosis (p = 0.02) compared to high LTA alone while VAN and DAP also increased phagocytosis but to a lesser extent in comparison to CFT and the increase did not differ significantly from LTA alone (p = 0.56, p = 0.51, respectively). With low level LTA exposure, VAN and CFT had no effects on phagocytosis, whereas DAP had a mild inhibitory effect that was not statistically significant.

### Neutrophil TLR2 cell surface expression

**Fig. 3:**
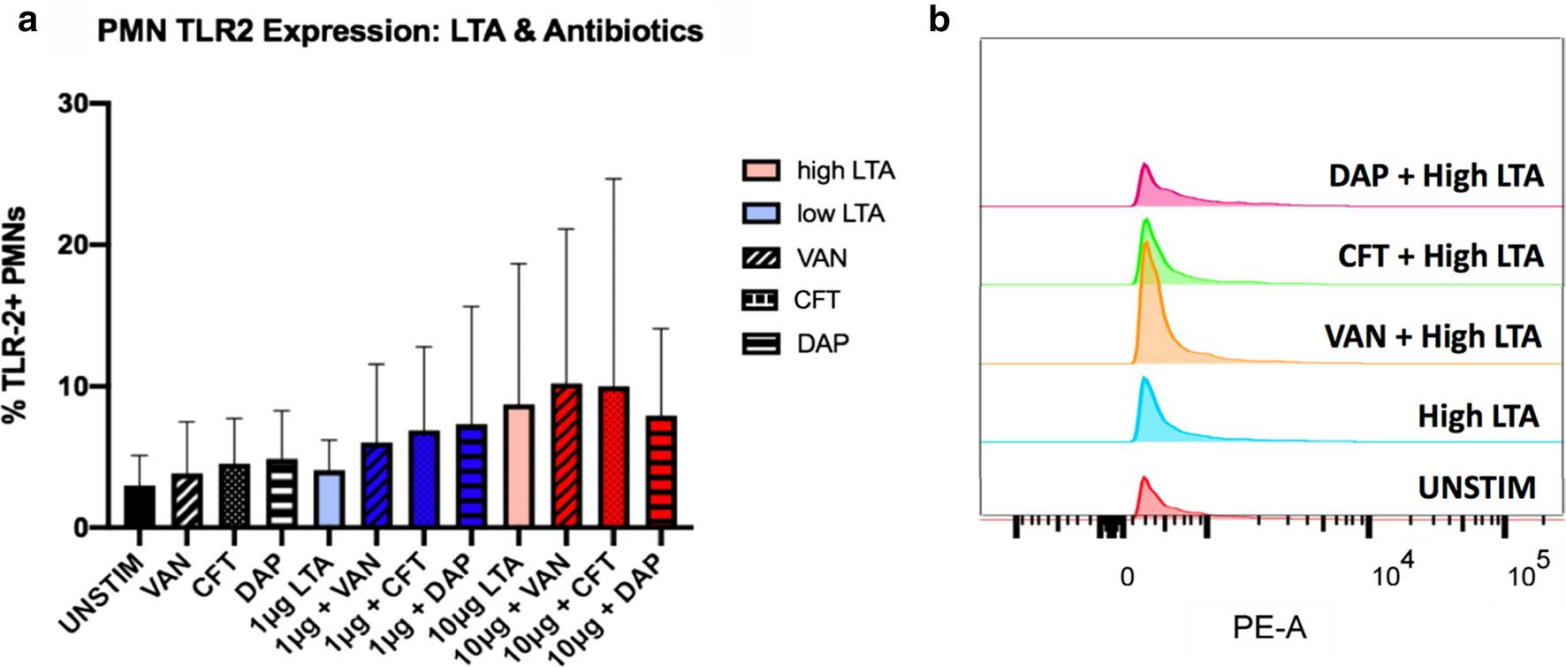
Effects of high (10 μg) and low (1 μg) levels of LTA and antibiotics on TLR-2 expression in human neutrophils isolated from healthy volunteer donors (n = 5). TLR-2 expression was determined at 4 h following stimulation using flow cytometry. Percentage of PE + cells is shown in (**a**). Representative histograms of PE fluorescence intensity in unstimulated cells and cells exposed to high LTA with and without antibiotics are shown in (**b**). Statistical significance was determined using Two-way ANOVA * p < 0.05. VAN, vancomycin; CFT, ceftaroline; DAP, daptomycin

**Fig. 4:**
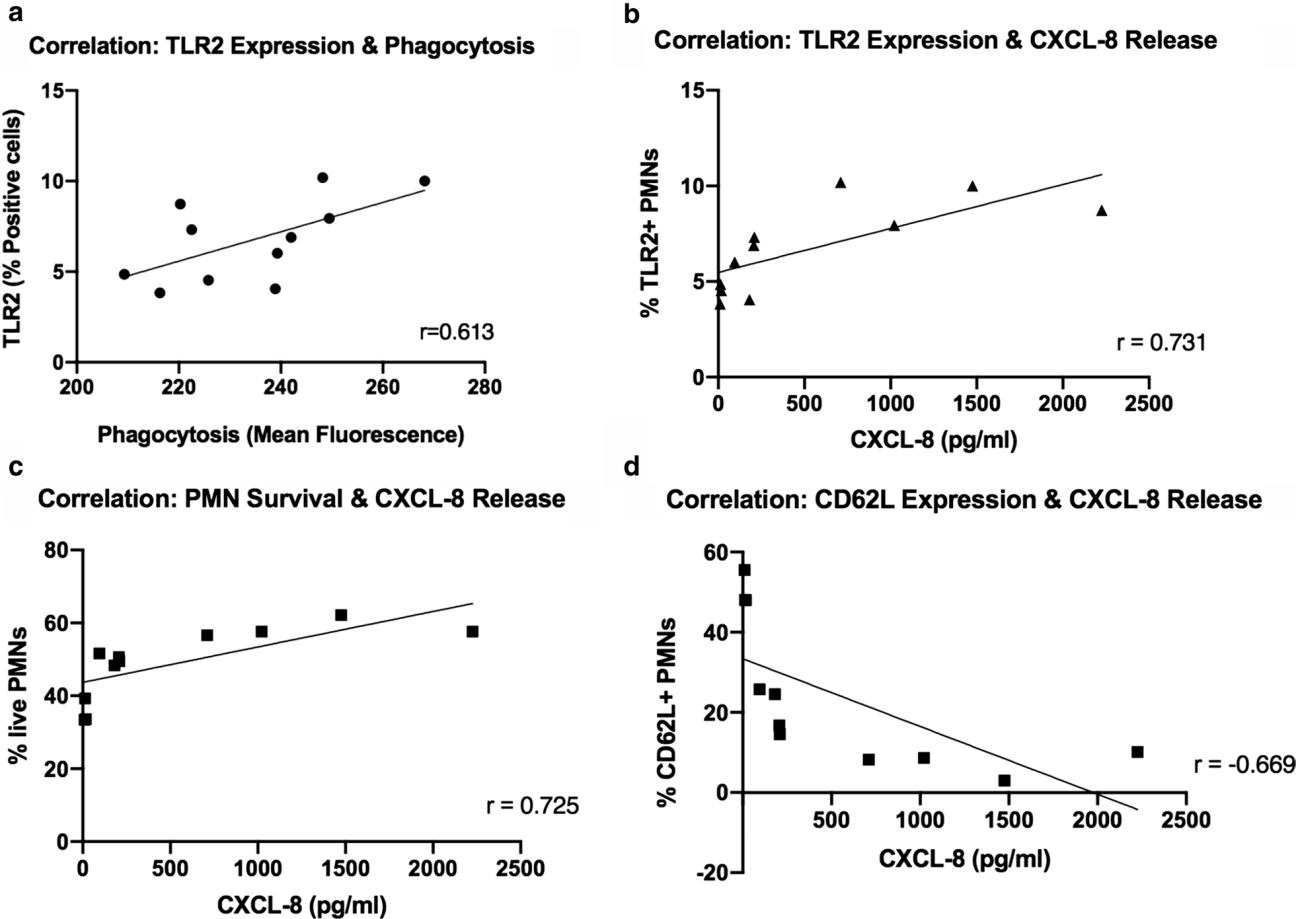
Correlation analysis of select variables: **a** TLR2 expression and phagocytosis; **b** TLR2 expression and CXCL8 release; **c** PMN survival and CXCL-8 release; and **d** CD62L expression and CXCL8 release. Correlation was determined using Pearson’s r value, as shown on each graph

PMN TLR2 cell surface expression was analyzed via flow cytometry, following 4 h of exposure to LTA with and without antibiotics (Fig. 3a, b). TLRs play a role in driving the inflammatory response with some studies suggesting that phagocytosis is a component of effective TLR2 signaling [30, 31]. While there were notable differences between the phagocytosis and TLR2 findings, TLR2 cell surface expression appeared to correspond with phagocytic function overall as determined by Pearson’s r correlation (p = 0.04) (Fig. 4a). In general, phagocytosis and TLR2 cell surface expression increase following exposure to high LTA levels. However, addition of antibiotics did not appear to directly alter TLR2 expression of PMNs in the presence of either high or low level of LTA.

### Neutrophil survival

PMNs have a short half-life in circulation if not activated by external stimuli, surviving for less than 24 h in the bloodstream [32]. PMN activation by LTA has been demonstrated to significantly lengthen lifespan, which may occur through NFκB signaling [16, 32]. In this study, PMNs were stimulated with or without LTA and antibiotics for 16 h. Following incubation, cells were harvested and stained with FITC-conjugated Annexin V and propidium iodide (PI). Annexin V binds to phosphatidylserine, a cell membrane component, which is located on the cell surface during apoptosis, whereas PI is a DNA-intercalating agent that is only able to permeate the cell membrane of dead cells. Double stained cells are considered to be in late apoptosis or necrosis [33]. Living cells, therefore, remain unstained by both Annexin V and PI.

**Fig. 5:**
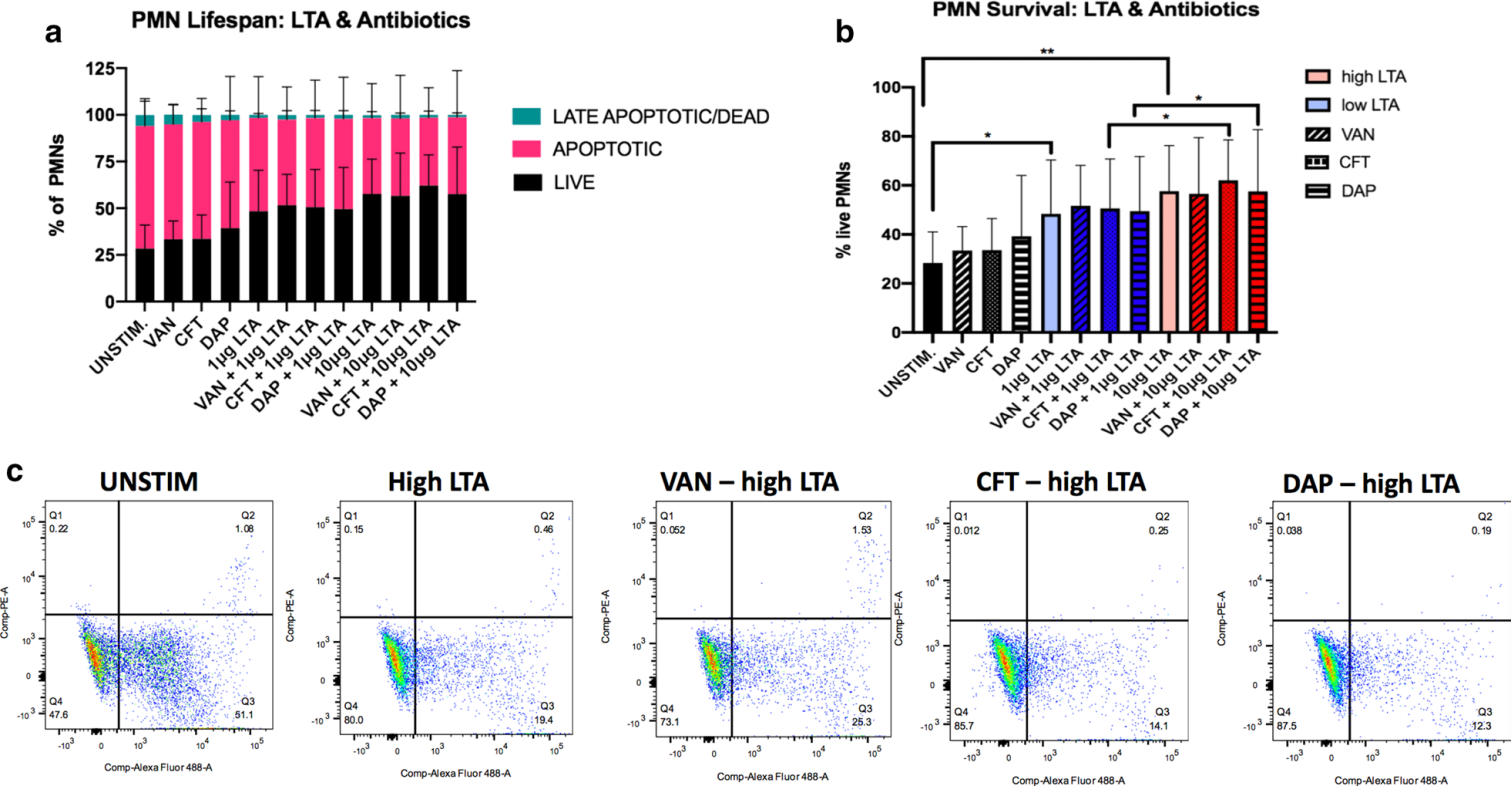
Effects of high (10 μg) and low (1 μg) levels of LTA and antibiotics on PMN lifespan (**a**) and survival (**b**) in human neutrophils isolated from healthy volunteers (n = 5). Effects on PMN lifespan and survival were determined at 16 h following stimulation using flow cytometry with Annexin V and propidium iodide (PI). Live cells are unstained, whereas Annexin V stains apoptotic cells and PI permeates dead cells. Double-stained cells are considered necrotic. Representative scatter plots of unstimulated cells and cells exposed to high LTA with and without antibiotics are shown in **c**. Statistical significance was determined using two way ANOVA * p < 0.05, **p < 0.01. VAN, vancomycin; CFT, ceftaroline; DAP, daptomycin

While cell death and necrosis were minimally observed across conditions, PMN apoptosis appeared to be dependent on stimulation with LTA (Fig. 5a–c). After 16 h, healthy donor PMNs that were not stimulated with LTA or antibiotics had a mean survival rate of 28.3% ± 12.70 (Fig. 3b). In comparison, PMNs stimulated with 1 μg/ml or 10 μg/ml LTA had mean survival rates of 48.3% ± 22.0 and 57.6% ± 18.5, respectively, which represents a significant increase in survival versus the unstimulated control (1 μg/ml, p = 0.01;10 μg/ml p = 0.008). Antibiotics alone, in the absence of LTA, had a modest, but not significant, positive effect on increasing PMN lifespan, in comparison to unstimulated PMNs (VAN, 33.42%; CFT, 33.60%; DAP, 39.30%). In the presence of high-level LTA, a trend towards prolonging PMN lifespan was observed with the addition of CFT (from 57.6% ± 18.59 to 62.1% ± 16.45, p = 0.08). VAN and DAP had modest positive effects on PMN survival. It is notable that significant differences in PMN survival were more likely detected between the different levels of LTA exposure than between different antibiotics, which supports LTA exposure level as the most likely factor contributing to the length of PMN lifespan.

### Vancomycin decreases CXCL8 release in PMNs under high exposure levels of LTA, whereas ceftaroline preserves pro-inflammatory response

**Fig. 6:**
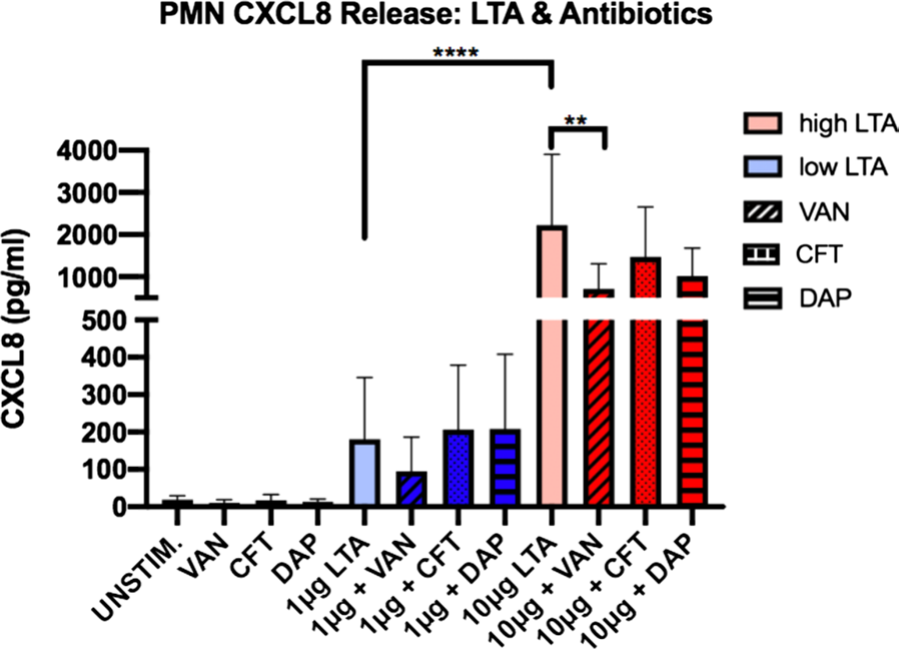
Effects of high (10 μg) and low (1 μg) concentrations of LTA and antibiotics on production of CXCL8 in human neutrophils isolated from healthy volunteers (n = 5). CXCL8 release was determined at 16 h following stimulation using ELISA. Statistical significance was determined using Two-way ANOVA. * p < 0.05, **p < 0.01, ****, p < 0.0001. VAN, vancomycin; CFT, ceftaroline; DAP, daptomycin

CXCL8 is a chemokine secreted by PMNs to stimulate a variety of pro-inflammatory innate immune responses, including phagocytosis, chemotaxis, and recruitment of additional PMNs to the site of infection [16, 34]. Following incubation for 16 h with or without LTA and antibiotics, PMN secretion of CXCL8 in cell culture supernatants was determined via ELISA (Fig. 6). The effect was demonstrated to be concentration-dependent, with high LTA causing significantly higher CXCL8 release in comparison to low LTA (p < 0.0001). In the presence of the high LTA, addition of VAN significantly decreased CXCL8 release (p = 0.005), and DAP showed a strong trend towards CXCL8 reduction (p = 0.06). Conversely, CXCL8 release was not significantly affected by addition of CFT, suggesting preservation of the pro-inflammatory response. In the presence of low LTA exposure, CFT and DAP had no effect on CXCL8 secretion from PMNs whereas a small, non-significant decrease was observed with VAN. Additionally, CXCL8 release was positively correlated with TLR-2 cell surface expression and cell survival, and negatively correlated with CD62L expression, based on Pearson r correlation (TLR-2, p = 0.01; survival, p = 0.01; CD62L, p = 0.02), which supports the role of CXCL8 in PMN activation and pro-inflammatory processes following exposure to LTA (Fig. 4b–d).

## Discussion

In this study, we evaluated antimicrobial agents commonly prescribed for the treatment of *S. aureus* bacteremia, including vancomycin, used as a standard treatment, and alternative agents such as daptomycin and ceftaroline; the latter are typically reserved for patients who have failed or are intolerant to vancomycin. Ceftaroline fosamil, is a pro-drug formulation of ceftaroline, approved by the FDA for the treatment of MRSA skin and soft tissue infections and community-acquired pneumonia. Though it is not specifically approved for use in bacteremia, it has been used successfully as salvage therapy in serious MRSA infections such as persistent bacteremia and infective endocarditis. We hypothesized that the therapeutic benefit observed with ceftaroline as salvage therapy during persistent bacteremia [22, 35, 36] may result from the innate immune stimulatory effects of ceftaroline, which have not been previously assessed. In this study, VAN and DAP demonstrated select, modest but insignificant stimulatory effects on PMNs in the presence of LTA (increased phagocytosis, increased CD11b production). Previous studies have shown that in the presence of the gram-negative endotoxin, lipopolysaccharide (LPS), VAN enhanced phagocytosis and gene expression of TLRs 1, 2, 4, and 7 in a human THP-1 monocytic cell line; however, VAN also induced IL-10 gene expression, as well as TNF and IL-1β response, indicating that it has pleiotropic effects on different cell types [37]. In a whole blood model of MRSA infection, VAN was shown to decrease IL-6 and CXCL8 release [38]; our study also demonstrated a reduction in CXCL8 release by VAN. Accordingly, VAN has also been shown to impact adaptive immunity in a study of patients with inflammatory bowel disease (IBD) in which VAN was shown to induce T regulatory cell production, which may have protective effects in the IBD population, but could negatively impact patients with sepsis by producing an excess of IL-10, contributing to the dysregulated immune response [39].

The immunomodulatory activities of DAP have also been assessed previously. Specifically, DAP was shown to decrease TLR 1, 2, and 6 expression and had no effect on phagocytosis [37]. Others have shown that in the presence of LPS, DAP has no significant effects on host cytokine response [40, 41]. Thallinger et al. speculated that DAP does not achieve high intracellular concentrations for different types of immune cells, including neutrophils [40]. Daptomycin is highly hydrophilic and has a very low volume of distribution, which may result in its limited capacity to penetrate effector immune cells and, subsequently, exert immunomodulatory effects by directly modulating human cellular or genetic factors [40, 42]. It is possible that the low volume of distribution as well as the hydrophilic core of daptomycin limits the drug’s ability to penetrate into the cytosol and nucleus necessary for exerting changes to host genetic regulatory mechanisms that control immune response [40, 43]. While the exact mechanisms that account for the differential immunomodulatory effects observed between antibiotics from different classes remain unknown, tight binding to intracellular structures or inactivation under low pH conditions inside the phagosome may reduce the intracellular bioactivity of one drug more than another [43–48]. Additional studies examining the underlying mechanisms of immunomodulatory activity, including variability in drug intracellular distribution in immune cells, are needed to better understand the complex interplay between host cells and antibiotics.

Our study demonstrates that in the presence of staphylococcal LTA, ceftaroline stimulates phagocytosis, cell survival, and PMN activation, as evidenced by its effects on CD11b and CD62L. Compared to vancomycin, which demonstrated dampening in CXCL8 release in the presence of high LTA, ceftaroline did not alter CXCL8 release, indicating a preservation of the pro-inflammatory response. The results of this study offer a preliminary biological rationale for the therapeutic benefit observed with ceftaroline in the setting of persistent *S. aureus* bacteremia through its immunostimulatory effects on neutrophils. While the immunomodulatory effects of ceftaroline in the presence of *S. aureus* components are not currently well-studied, several studies have reported an association between extended ceftaroline exposure and neutropenia in patients with a variety of different infections [49, 50]. While the relationship between enhanced PMN activation and the incidence of neutropenia is unclear and has not been studied to date, it is possible that in the setting of prolonged ceftaroline exposure, the observed activating effect could lead to eventual PMN exhaustion and depletion. As has been demonstrated previously, persistently active PMNs adopt an “exhausted” phenotype, marked by ineffective bacterial clearance and increased expression of immunosuppressive markers [51]. Exhausted cells are prone to dramatic changes in phenotype, as well as re-migration into the bone marrow to undergo apoptosis [52]. This hypothesis, as well as the other findings reported in this study, deserve further investigations by additional in vivo and clinical studies in patients with persistent bacteremia.

While LTA remains an important gram-positive virulence factor, which is present in all Gram positive bacteria, *S. aureus* contains an arsenal of other important virulence factors that may differentially influence the host immune response. Further studies are warranted to understand the effect of antibiotics in the presence of a variety of virulence factors, including to use of live clinical bacterial strains, to mimic in vivo infection. Studies of other virulence factors and *S. aureus* components have demonstrated differential immunomodulatory effects of antibiotics. For example, Franks et al. showed that linezolid is more potent than vancomycin in suppressing pro-inflammatory cytokine production and that the effect diminishes when addition of the antibiotic to monocytes infected with MRSA ex vivo is delayed from 3 to 9 h [53]. Pichereau et al. found daptomycin to inhibit production of pro-inflammatory cytokines in monocytes after exposure to *S. aureus* toxins such as Panton-Valentine leukocidin and alpha-toxin [41]. In addition to direct immunomodulatory effects, antibiotics may also directly impact *S. aureus* virulence factor expression, which in turn may indirectly influence the host immune response. For example, ceftaroline has been found to affect *S. aureus* adhesion gene regulation, whereas other beta-lactam drugs have been shown to promote mRNA expression of Panton Valentine Leucocidin (PVL), a hemolytic toxin common in *S. aureus* clinical strains [54, 55]. Similarly, protein synthesis inhibitors have been shown to modulate production of phenol soluble modulins, toxins which attract human PMNs via the human formyl peptide receptor 2, and affect the innate immune response [56, 57].

Taken together, the results of our study may provide initial support towards the selection of therapies for the treatment of *S. aureus* bacteremia by harnessing the immunomodulatory potential of different antibiotic agents, though these results must be further validated with a larger clinical study in patients with SAB. If clinical data supports the preclinical ex vivo findings of antibiotic immunomodulatory activity, agents such as vancomycin and daptomycin could be selected as treatment options for their “neutral” effects on innate immunity, which would be beneficial for patients with a relatively “balanced” pro-inflammatory and anti-inflammatory immune response. These patients may have better outcomes with therapies that minimally interfere with the natural course of the immune response, as not all patients are suitable candidates for agents that enhance pro-inflammatory response. Conversely, ceftaroline could be beneficial in patients with persistent bacteremia resulting from a dysregulated immune response, given the drug’s activating effect on neutrophil function. Future studies of anti-staphylococcal therapeutics should examine the mechanism of action to determine how antibiotics from different pharmacologic classes interact with host immune cells to exert immunomodulatory activity besides their direct antibacterial activity.

## Conclusion

The findings from this study demonstrate that antibiotics have differential immunomodulatory effects on human neutrophils stimulated with two unique exposure levels of LTA. Ceftaroline, in particular, has robust stimulatory effects on neutrophil phagocytosis and activation, which has not been assessed previously, whereas vancomycin decreases CXCL8 release and daptomycin exerts minimal effects on neutrophils overall. These observations suggest that therapy selection should take into account bacterial burden, strain-specific differences in LTA production, and host immune status as initial steps towards implementation of precision therapies for infectious diseases. Predictive modelling experiments of SAB incorporating patient data such as genotypes and clinical outcomes, have demonstrated the advantage of precision therapies for SAB [58]. Since antibiotics are the primary therapy used to treat SAB, they present a unique opportunity to harness both direct antibacterial and immunomodulatory activity as a comprehensive strategy to individualize treatment for persistent *S. aureus* bacteremia.

## Data Availability

The data used in this study are available upon reasonable request to the corresponding author.
